# Supplementary material for: Localization of seismic waves with submarine fiber optics using polarization-only measurements
Source: Commun Eng. 2023 Dec 4;2:86. doi: 10.1038/s44172-023-00138-4 (PMC10955929; doi:10.1038/s44172-023-00138-4)
Supplement: Supplementary file 2 — Supplementary Information [file 44172_2023_138_MOESM2_ESM.pdf]

Localization of seismic waves with submarine fiber optics using polarization-only measurements: Supplemental Document

SUPPLEMENTARY NOTE 1

Data processing: Eigenvalue method

The following processing was used to recover local earthquake data using the eigenvalue method:

For every pulse launched into the fiber, M reflections are recorded corresponding to the number of repeaters in the cable. An acquisition (at time t) comprises every set of 3 consecutive measurements (obtained from probing the fiber with three different input SOP). By storing the recovered normalized stokes vectors from each m-th repeater as the columns of a 3x3 matrix, we are able to construct the matrices that encode the matrices $\mathbf{A}_m(t)$

After constructing the $\mathbf{A}_m(t)$ matrices from measurements, we perform the singular value decomposition of each to compute the closest unitary matrix to $\mathbf{A}_m(t)$, $\hat{\mathbf{A}}_m(t) = \mathbf{U}\mathbf{V}^{\mathrm{T}}$ as a denoising step.

The local birefringence matrices ($\mathbf{X}_m(t)$) are then calculated using the measured (and denoised) cumulative matrices. The eigenvalues of this matrix are then calculated, which are of the form $1, \exp(+i\theta), \exp(-i\theta)$. We keep the one with the positive argument ($\exp(+i\theta)$) and measure its difference with the first acquisition.

The final output is a matrix of M columns and T rows, where T is the total number of acquisitions. The processing steps are depicted in figure S1.

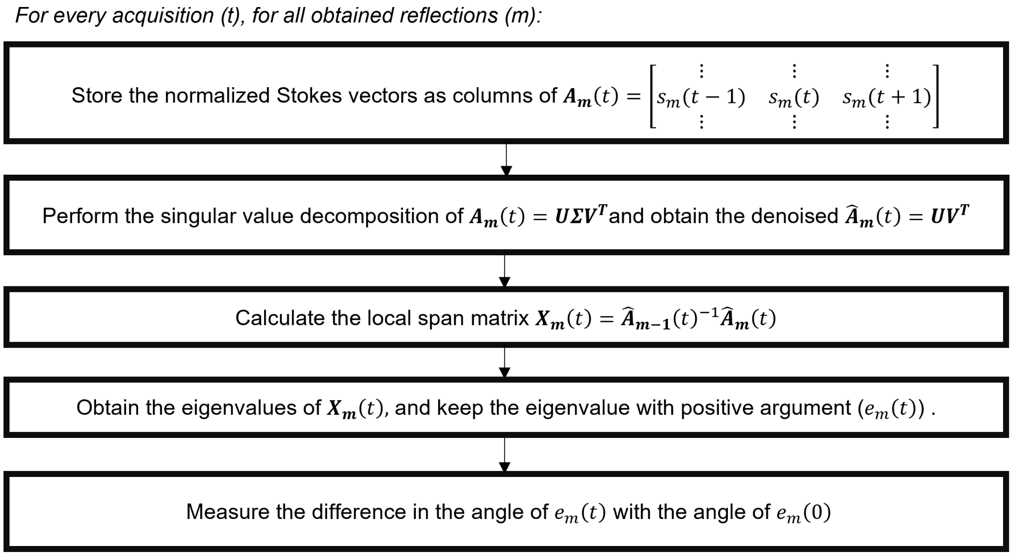

Fig. S1. Eigenvalue processing stack. Visual depiction on how to recover the local time series using the eigenvalue method. t is an index referring to each acquisition and m is an index corresponding to each span or repeater.

Data processing: Direct SOP method

The direct SOP method consists of probing the fiber repeatedly with the same input SOP, and recording changes to each of the three Stokes components of the output normalized Stokes vectors. As such, this method produces three output time-series, one for each of the output S components.

For the input SOP, the input SOP must remain stationary, which is not the case in our experiments (the SOP of probe pulses is being cycled through a set of three Stokes vectors). As such, we split the data into three datasets to be processed independently each corresponding to one of the three possible input SOP. Each of these datasets produces itself three output S components, resulting in a total of nine independent measurements.

When performing the direct SOP method we may or may not calculate the difference with the previous repeater. In both cases, the measurements are not localized. In our experiments we did not calculate the difference with the previous repeater. For the 2D plot of Figure 3 in the main text, the nine measurements were processed independently to generate a 2D plot, and all 9 resulting 2D plots were combined by averaging.

SUPPLEMENTARY NOTE 2

Acquisition rate

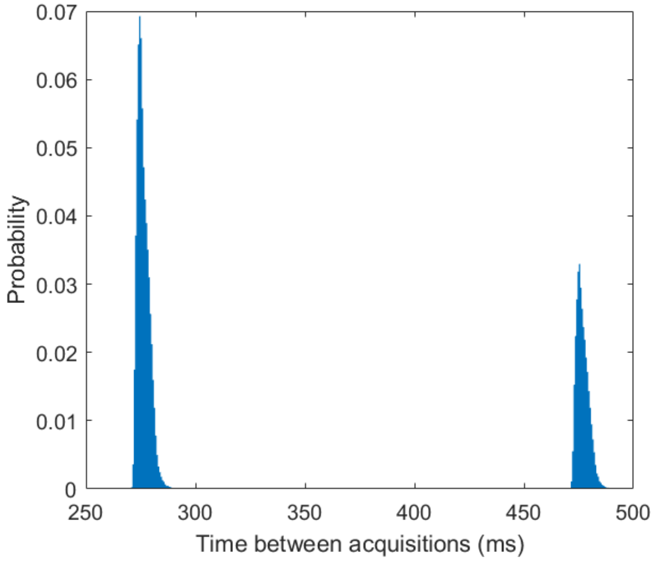

Fig. S2. Acquisition rate. Histogram of time interval between acquisitions over 24 hours, demonstrating the longer delay occurring every third pulse.

In the field demonstration presented in the main text, the pulse repetition rate is adjusted to align with the fiber length constraint (calculated as 105 ms using equation 6). However, the PSY-201 polarization synthesizer can only accommodate voltage change commands (V1 and V2) every 200 ms. This constraint means that the pulse triplet cannot be efficiently generated by controlling the polarization of each individual pulse, necessitating the selection of probe pulses with the appropriate modulation.

We generate the input basis according to the following procedure, assuming the initial probe state is s_1:

- Capture s_1 SOP at t = 75 ms and update V1 at t = 275 ms.
- Capture s_2 SOP at t = 350 ms and update V2 at t = 550 ms.
- Capture s_3 SOP at t = 625 ms, then sequentially update V1 at t = 825 ms and V2 at t = 1025 ms.
- Capture s_1 SOP again at t = 1100 ms and update V1 at t = 1300 ms.

Note that the delay between the first and second captures and between the second and third captures is fixed at 275 ms. However, a longer delay of 475 ms occurs between the third and fourth captures, resulting in non-uniform sampling. This can be seen in the histogram of acquisition intervals in Figure S2.

SUPPLEMENTARY NOTE 3

Q Measurements

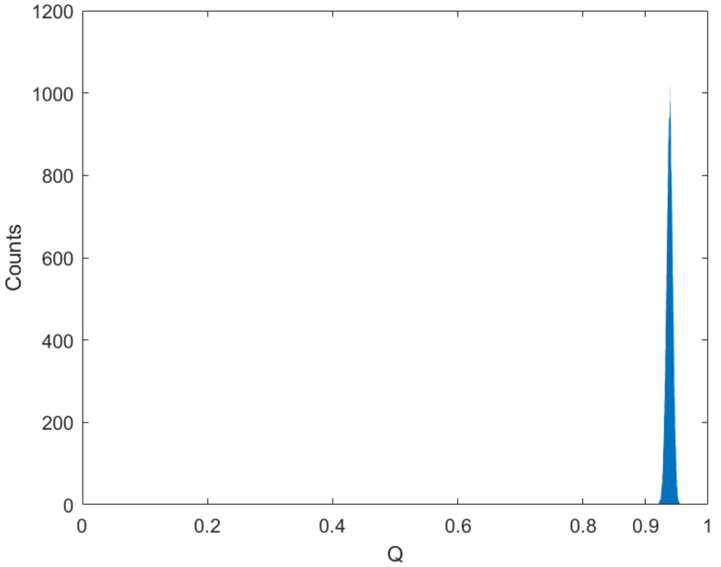

Fig. S3. Histogram of Qs. Histogram registered over 24 hours, where Q is shown to be kept consistently above 0.9.

In principle, the eigenvalue method can operate with any trio of input SOPs that spans the entire Stokes space. However, it is advantageous to use an orthogonal basis for optical noise robustness and mitigation of crosstalk.

One possible figure of merit (FOM) for the orthogonality of the selected input basis can be

$$Q = |\det(\mathbf{A}_0)|, \tag{S1}$$

where $\mathbf{A}_0$ is a 3x3 matrix where each column is the normalized Stokes vector of each of 3 consecutive input SOP. Q varies between 0 for a set of three input vectors that do not form a basis of the full Stokes space, to 1 where the set of vectors forms an orthonormal basis.

We monitored the Q of each acquisition by measuring the 3 polarization states launched into the fiber at every acquisition, and kept the Q consistently above 0.9. The histogram of measured Q values is displayed in figure S3

SUPPLEMENTARY NOTE 4

Crosstalk and nonlocal effects

We performed a numerical evaluation of the effects of different perturbation parameters and input triplets on the observed crosstalk. The simulations comprise a simulated cable composed of N spans, each including (independent) forward and backward paths. Each path has a random birefringence vector orientation with a fixed, pre-determined birefringence strength. The fixed simulation parameters are represented in table S1.

In order to assess the onset of crosstalk and nonlocal effects when using the eigenvalue method, we added a sinusoidal variation of 0.02 percent to both the forward and backwards paths of the second span in the cable.

We repeated the measurement for the same cable while varying the input basis set to have different Q (equation S1) and changing the frequency of the sinusoidal perturbation (while keeping the acquisition rate and amplitude fixed). Altering the perturbation frequency results in a more significant waveform change over the three acquisitions needed for a complete measurement of the $\mathbf{A}(m)$ matrix, thus leading to a stronger breach of the stationarity assumption. For this simulation, we considered infinite optical SNR.

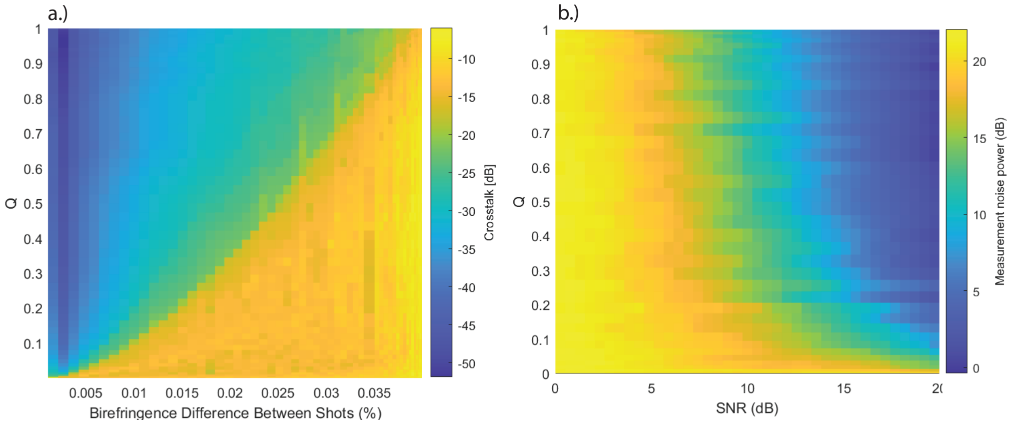

Fig. S4. Numerical simulation results. Simulated results of crosstalk observed as a function of input Q and maximum perturbation slew rate. Crosstalk is measured as the median variance measured in spans after the perturbed span, normalized to the variance of the perturbed span.

**Parameter**	**Value**
Number of Spans	40
Span length	100 km
Average birefringence strength (Δn)	1×10^{-8}
Perturbation amplitude (change in Δn)	0.02% of birefringence
Perturbed span	2

Table S1. Summary of Simulation Parameters

We define crosstalk as the median variance of the signal observed in all (unperturbed) spans located after the perturbed span, normalized to the variance of the signal observed in the perturbed span (which may change between runs of the simulation, due to the nonlinearity of eigenvalue measurements). In figure S4a, we plot the crosstalk against the orthogonality figure of merit (Q) and the maximum birefringence change between consecutive acquisitions (as a measure of non-stationarity).

There is a clearly observable a threshold effect where the crosstalk suddenly increases by about 10 dB, for combinations of low Q and high slew-rate. Two possible ways to improve or mitigate crosstalk are to ensure consistently high Q and orthogonality, as well as maximizing the acquisition rate of the system. This also implies that the amount of crosstalk to subsequent spans is frequency dependent. For fixed amplitude, higher frequency perturbations may lead to increased crosstalk to subsequent positions, which also suggests that simple low-pass filtering of all channels may be sufficient to reduce crosstalk for some applications.

We also observed the effects of Q and optical SNR on the measurement noise power, as depicted in figure S4b. Higher Q and higher SNR seem to correlate with lower measurement noise. Our simulations seem to suggest that there is a minimum value of Q (around 0.3) at which robustness to low optical SNR increases.
